# COVID-19: lessons for junior doctors redeployed to critical care

**DOI:** 10.1136/postgradmedj-2020-138100

**Published:** 2020-06-24

**Authors:** Charles Coughlan, Chaitanya Nafde, Shaida Khodatars, Aimi Lara Jeanes, Sadia Habib, Elouise Donaldson, Christina Besi, Gurleen Kaur Kooner

**Affiliations:** Cardiac Intensive Care Unit, Hammersmith Hospital, Imperial College Healthcare NHS Trust, London, UK; Department of Primary Care and Public Health, Imperial College London, London, UK; Cardiac Intensive Care Unit, Hammersmith Hospital, Imperial College Healthcare NHS Trust, London, UK; Cardiac Intensive Care Unit, Hammersmith Hospital, Imperial College Healthcare NHS Trust, London, UK; Cardiac Intensive Care Unit, Hammersmith Hospital, Imperial College Healthcare NHS Trust, London, UK; Cardiac Intensive Care Unit, Hammersmith Hospital, Imperial College Healthcare NHS Trust, London, UK; Cardiac Intensive Care Unit, Hammersmith Hospital, Imperial College Healthcare NHS Trust, London, UK; Cardiac Intensive Care Unit, Hammersmith Hospital, Imperial College Healthcare NHS Trust, London, UK; Cardiac Intensive Care Unit, Hammersmith Hospital, Imperial College Healthcare NHS Trust, London, UK

**Keywords:** infectious diseases, adult intensive & critical care, medical education & training, respiratory infections

## Abstract

Approximately 4% of patients with coronavirus disease 2019 (COVID-19) will require admission to an intensive care unit (ICU). Governments have cancelled elective procedures, ordered new ventilators and built new hospitals to meet this unprecedented challenge. However, intensive care ultimately relies on human resources. To enhance surge capacity, many junior doctors have been redeployed to ICU despite a relative lack of training and experience. The COVID-19 pandemic poses additional challenges to new ICU recruits, from the practicalities of using personal protective equipment to higher risks of burnout and moral injury. In this article, we describe lessons for junior doctors responsible for managing patients who are critically ill with COVID-19 based on our experiences at an urban teaching hospital.

## Introduction

The COVID-19 pandemic has caused unprecedented disruption to health services worldwide. Approximately 4% of patients with confirmed COVID-19 become critically ill and require admission to the intensive care unit (ICU) for respiratory support.^1^ Early ICU audit data from the UK suggest that 25%–30% of these patients develop multiorgan failure, requiring additional cardiovascular and/or renal support.^2^ This global public health crisis poses major operational and logistical challenges, even in high-income countries with well-resourced health systems.^3^ Hospitals and regional and national governments have scrambled to increase capacity, source vital equipment and redistribute staff to meet rising demands for critical care. In the UK, junior doctor rotations have been cancelled and many doctors in training with no or minimal experience of ICU medicine have been redeployed to work in this demanding specialty.^4^

Under normal circumstances, ICU medicine can be daunting for junior doctors. Notable challenges include managing multiple unwell patients simultaneously and different patterns of working alongside highly skilled nursing and ancillary staff. In the context of a pandemic, doctors working in critical care may experience additional strain as a result of staff absenteeism,^5^ high workload and moral injury.^6^ Nonetheless, junior doctors are motivated to contribute to the COVID-19 pandemic response and with support from senior staff can contribute to high-quality patient care and reap educational rewards. We have adapted to work in challenging circumstances in a COVID-19 ICU in an urban teaching hospital. This article aims to provide junior doctors redeployed to ICU in the COVID-19 pandemic with insights from our collective experience.

### ICU management

Patients who are critically ill can deteriorate rapidly, and intensive care calls for detailed, thorough and repeated clinical assessments. A structured and systematic clinical assessment (such as FAST HUGS BID) ensures that pressing issues are identified and addressed promptly.^7^ One of the key differences between ward and intensive care medicine is the intensity of multidisciplinary team (MDT) working.^8^ ICU nurses are highly skilled; they are comfortable with complex equipment, manage and titrate multiple intravenous infusions and typically care for just one to two patients per shift.^9^ Most patients who are critically ill require nasogastric feeding or parenteral nutrition and dieticians regularly optimise their feeding regimes. Physiotherapists play a key role in rehabilitation, notably in managing respiratory weaning after tracheostomy insertion.^10^ Speech and language therapists support the restoration of voice and swallow after critical illness.^11^ This MDT approach ensures that patients receive tailored and holistic care and is central to good clinical outcomes.^8^

Critical care medicine requires close attention to detail and responsiveness to sudden changes in physiology and blood test results. Detailed ICU management of COVID-19 is described elsewhere.^12^ We will outline several salient aspects of day-to-day management relevant to non-airway-trained doctors redeployed to ICU. While local, regional and national guidelines may vary, patients who are critically ill with COVID-19 typically receive a short course of empirical antibiotics.^13^ This reflects the practical difficulty of distinguishing between primary viral infection and bacterial sepsis and the potential for superadded bacterial infection. If available, procalcitonin can help guide antibiotic treatment in febrile patients. This biomarker—more specific for bacterial infection than C-reactive protein^14^—has been shown to reduce unnecessary antibiotic prescribing in critical care.^15^ However, ICU patients have many potential sources of fever. Regular consultation with specialist microbiology teams is therefore warranted to optimise antibiotic prescribing, particularly in patients with prolonged ICU stays.

Cross-sectional imaging may be necessary to exclude differential diagnoses such as pulmonary embolism, but this is complicated by logistical challenges including transfers by anaesthetic staff and use of a dedicated machine. Junior staff can streamline imaging by liaising closely with radiologists, radiographers and anaesthetists. Finally, several studies have highlighted coagulopathy in severe COVID-19 and specialists have advocated aggressive anticoagulation strategies based on D-dimer results.^16^ With the current evidence gap, different ICUs are likely to adopt different anticoagulation protocols. Nonetheless, for junior doctors, daily monitoring of D-dimers and close consultation of anticoagulation guidelines is essential, particularly for patients with renal impairment.

### Interprofessional communication

The detail and complexity of critical care necessitates clear communication within the medical team and between doctors, nurses and allied health professionals. Visual aids including anonymised whiteboards displaying physiological parameters, test results and ceilings of care can maintain effective communication between shifts and within teams with high staff turnover.

Infection prevention and control measures will lead to separation of team members in ‘clean’ and ‘dirty’ areas. Division of staff increases the risk of delays in the recognition, assessment and treatment of patients who may deteriorate rapidly. We have used remote communication aids including teleconferencing software and walkie-talkies in an attempt to bridge the gap between doctors providing direct care and their colleagues in ‘clean’ areas. This inevitably prolongs ward rounds and risks communication errors, which can be minimised by uninterrupted verbal handover and ‘safety huddles’.^17^ In our experience, this is particularly important in COVID-19 due to the subtle but important differences between patients with similar disease phenotypes.

Two weeks into our pandemic response, we transitioned to a fully integrated electronic patient record. We had previously used a different electronic record for drug prescribing and medical notes, alongside bedside paper nursing charts. Initially, our new system caused some disruption to workflow, particularly for nursing staff responsible for inputting patient-level data. However, a fully integrated record has enabled medical staff and allied health professionals to rapidly review a patient’s physiological parameters, drug chart and progress notes to make informed remote decisions about patient management. We have continued to use laminated bedside charts displaying daily medical management plans to reinforce key messages during this transition period.

### Communicating with relatives

Friends and relatives can play an important role in the rehabilitation of patients who are critically ill by helping to prevent delirium^18^ and reduce patient anxiety.^19^ Visits also allow doctors and nurses to obtain a collateral history and glean insights into the patient’s functional status and family dynamics. However, in the UK and many other countries, hospital visits have been banned to prevent onward severe acute respiratory distress syndrome coronavirus 2 (SARS-CoV-2) transmission. Relatives are understandably concerned, and we therefore aim to provide a daily telephone update to the patient’s next of kin. This difficult task usually falls to junior doctors. Even with face-to-face meetings, patient representatives often misunderstand the diagnosis, prognosis and treatment plans for patients who are critically ill.^20^ High staff turnover in ICU can also lead to inconsistent communication with relatives.^21^

We keep track of daily discussions using a dedicated whiteboard and have used structured communication aids including an infographic developed by the palliative care team at an urban district general hospital^22^ to promote consistent and compassionate telephone communication with families. In our experience, conversations around ceilings of care, unexpected deterioration and end-of-life care can be particularly distressing for relatives and challenging for doctors. We have used teleconferencing tools on tablet and mobile computers to enable relatives to see and pray for patients at the end of their lives. Junior doctors can facilitate these calls to relieve overstretched nursing staff and explain the ICU environment to relatives.^23^

### Physical and mental health

Healthcare workers are at increased risk of acquiring SARS-CoV-2 infection^24^ and developing mental health problems due to severe stress.^6^ Although most junior doctors are at low risk of severe COVID-19, many are understandably concerned about falling ill.^25^ Several members of our team who developed symptoms or had to self-isolate due to illness among household contacts felt guilty at staying at home while colleagues continued to work in challenging circumstances. They also reported concern at the possibility of clinical deterioration after a positive test result or onward transmission to vulnerable relatives. Different pathways exist within and between countries for testing healthcare workers, with ICU staff usually prioritised. We recommend contacting your line manager at the earliest opportunity if unwell in order to gain prompt access to testing.

In the 2003 SARS outbreak, correct use of personal protective equipment (PPE) significantly reduced the risk of infection among healthcare workers.^26^ However, wearing PPE for prolonged periods is hot and uncomfortable and causes difficulties in performing even basic procedures such as blood sampling and cannulation. Self-contamination is most common during PPE doffing and evidence suggests that this can be reduced by using a buddy to identify and rectify errors in technique and using standardised written protocols.^27^ New guidance from professional bodies emphasises the importance of donning adequate PPE before commencing assessment and treatment^28^ and junior doctors must resist the temptation to rush to emergencies without suitable protection.

ICU is a surreal world in the COVID-19 pandemic, with redeployed doctors working for long periods on demanding rotas. Absenteeism due to ill health and childcare commitments can lead to unsustainably high workloads in ICU during pandemics.^29^ Severe illness within clinical teams can cause additional stress and premature deaths of colleagues can severely impair morale.^30^ Redeployed doctors are already experiencing significant upheaval and may experience additional feelings of anxiety, helplessness, shock and grief in such an intense environment. Simple measures, such as providing access to ‘Wobble rooms’ (places for staff to unwind after dealing with difficult situations) and establishing junior doctor forums similar to Schwartz rounds, can safeguard mental well-being in redeployed doctors.^31^ However, senior staff must be mindful that signposting well-being resources to junior staff will not necessarily lead to them accessing appropriate help.^6^

### Working outside competencies and cross-skilling

In the UK, professional bodies have acknowledged that doctors must be prepared to work beyond their usual competencies in the pandemic response. Redeployed doctors may be concerned about negligence proceedings resulting from work in an unfamiliar setting.^32^ US states including New York have enacted emergency legislation to protect doctors from future civil cases resulting from injuries or deaths during the COVID-19 pandemic.^33^ The UK General Medical Council has issued reassurance that complaints made against doctors acting outside their normal role will be considered in light of an unprecedented public health crisis.^34^ The risk of poor patient outcomes can be mitigated by comprehensive local induction^35^ and this must be prioritised by senior clinicians despite operational pressures.

The Faculty of Intensive Care Medicine has created an online guide to support healthcare providers seeking to safely redeploy non-ICU-trained healthcare professionals to critical care.^36^ This guide, endorsed by the Royal College of Surgeons, emphasises that doctors with no prior training will not be expected to perform intubation or advanced airway manoeuvres. However, all doctors have a range of transferable skills relevant to critical care, from data interpretation to practical procedures such as peripheral cannulation and catheterisation. In our tertiary centre, cardiothoracic surgery specialty trainees have assisted with routine but essential tasks including proning, washing and turning patients, drawing up and administering medications and arterial blood gas sampling. Intensive care outcomes are highly sensitive to nurse to patient ratios^37^ and by assisting with nursing duties under appropriate supervision, redeployed doctors can mitigate the impact of absenteeism on patient care. Like us, junior doctors may also gain a renewed appreciation for the hard work performed by our highly skilled nursing colleagues.

It is important to remember that your colleagues may have been redeployed to critical care from academic or community-based roles. They may take time to adjust to an intense new environment. We all have the same goal—to achieve the best outcomes for our patients. Kindness and support can go a long way in helping your colleagues during a challenging time.

### Education and Training

The COVID-19 pandemic has significantly disrupted education and postgraduate training for junior doctors. Face-to-face teaching and postgraduate examinations^38^ have been cancelled in the UK due to operational constraints, infection prevention and control guidance, and social distancing legislation. This has led to anxiety and apprehension among junior doctors, exacerbated by uncertainty over future clinical attachments and working patterns. In the UK, statutory education bodies have issued contingency plans for more flexible annual appraisals which will take account of time off due to illness and evidence of progression.^39^

The pandemic has catalysed a rapid shift towards online medical education.^40^ Virtual Grand Rounds have been launched at our multisite hospital Trust, and Foundation Teaching will soon be delivered by teleconferencing. With busy rotas and significant workloads, it may be difficult for junior doctors working in critical care to access formal teaching sessions online. However, ICU is a rich learning environment and with support, redeployed junior doctors can become more confident in managing unwell patients and performing advanced practical procedures including arterial and central line insertion.

Where possible, departmental teaching can and must shift to focus on areas relevant to COVID-19. Training in our Trust for redeployed doctors (including middle-grade doctors already specialising in anaesthesia and intensive care) has included modified intubation protocols; basics of mechanical ventilation and ventilator troubleshooting; and use of inotropes and sedation. Early work suggests that in situ simulation for staff responsible for managing patients who are critically ill with COVID-19 can identify safety issues and optimise workflow.^41^ In our experience, high-yield simulation topics include safe donning and doffing of PPE, proning and modified cardiopulmonary resuscitation.

Junior doctors redeployed to ICU can also contribute to patient care through audit and quality improvement. In response to a clinical incident in which a patient’s planned tracheostomy procedure was delayed by inappropriate anticoagulant administration, members of our team have developed a preoperative surgical tracheostomy checklist. This has been enthusiastically adopted by our specialist surgical teams and expanded to cover other ICU sites at two other hospitals within our multicentre National Health Service Trust. Quality improvement initiatives have also led to the development of similar checklists for intubation and proning. Frequent changes in clinical guidelines for COVID-19 management in critical care can make audit and quality improvement challenging. We recommend that trainees focus on core processes including completeness of daily medical assessments and communication with patient families.

## Discussion

We have outlined six key lessons relevant to junior doctors redeployed to critical care during the COVID-19 pandemic. These are based on our collective experiences in an ICU at an urban teaching hospital within a multihospital Trust serving a diverse patient population.^42^ These reflections come from doctors working in a high-income country with a universal healthcare system. This may reduce their relevance to doctors working in resource-limited settings.

We recommend that senior clinicians and managers listen carefully to redeployed doctors’ concerns and where possible arrange shadowing and simulation training prior to redeployment. Given the high mortality seen in patients with COVID-19 admitted to ICUs, and communication challenges posed by strict limitations on family visits, junior doctors should receive additional training and support in breaking bad news. Well-being is particularly important for redeployed doctors and simple measures such as introducing junior doctor forums can provide trainees with a space to reflect on stressful experiences with their peers. Future research should assess the impact of redeployment on postgraduate outcomes, burnout and morale among junior doctors.

Despite considerable disruption to postgraduate training and education, redeployment to critical care offers unique opportunities for clinical and professional development. Senior support can help junior doctors acquire transferable skills that will enhance their performance in any field of medicine.

**Main messages**

- In a pandemic setting, many junior doctors with little or no experience of critical care can expect to be redeployed to intensive care unit to enhance surge capacity.
- Although redeployment may disrupt training, teaching and postgraduate examinations, critical care is a rich learning environment for junior doctors.
- Senior colleagues should pay particular attention to junior doctor well-being, simulation training and interprofessional communication.

**Current research questions**

- Are there differences in mental health outcomes among junior doctors redeployed to critical care and their colleagues working in other specialties during the COVID-19 pandemic?
- Have junior doctor perceptions of critical care changed following redeployment?
- To what extent has redeployment to critical care during the COVID-19 pandemic disrupted career progression for junior doctors?
